# Extended Joint Sparsity Reconstruction for Spatial and Temporal ERT Imaging

**DOI:** 10.3390/s18114014

**Published:** 2018-11-17

**Authors:** Bo Chen, Juan F. P. J. Abascal, Manuchehr Soleimani

**Affiliations:** 1 Engineering Tomography Lab (ETL), Department of Electronic and Electrical Engineering, University of Bath, Bath BA2 7AY, UK; B.Chen@bath.ac.uk; 2 Univ Lyon, INSA-Lyon, Université Claude Bernard Lyon 1, UJM-Saint Etienne, CNRS, Inserm, CREATIS UMR 5220, U1206 Lyon, France; juanabascal78@googlemail.com

**Keywords:** electrical resistance tomography, total variation (TV) algorithm, dynamical ERT

## Abstract

Electrical resistance tomography (ERT) is an imaging technique to recover the conductivity distribution with boundary measurements via attached electrodes. There are a wide range of applications using ERT for image reconstruction or parameter calculation due to high speed data collection, low cost, and the advantages of being non-invasive and portable. Although ERT is considered a high temporal resolution method, a temporally regularized method can greatly enhance such a temporal resolution compared to frame-by-frame reconstruction. In some of the cases, especially in the industrial applications, dynamic movement of an object is critical. In practice, it is desirable for monitoring and controlling the dynamic process. ERT can determine the spatial conductivity distribution based on previous work, and ERT potentially shows good performance in exploiting temporal information as well. Many ERT algorithms reconstruct images frame by frame, which is not optimal and would assume that the target is static during collection of each data frame, which is inconsistent with the real case. Although spatiotemporal-based algorithms can account for the temporal effect of dynamic movement and can generate better results, there is not that much work aimed at analyzing the performance in the time domain. In this paper, we discuss the performance of a novel spatiotemporal total variation (STTV) algorithm in both the spatial and temporal domain, and Temporal One-Step Tikhonov-based algorithms were also employed for comparison. The experimental results show that the STTV has a faster response time for temporal variation of the moving object. This robust time response can contribute to a much better control process which is the main aim of the new generation of process tomography systems.

## 1. Introduction

Electrical resistance tomography has been investigated for few decades since it was proposed in 1984 as an approach for vivo image reconstruction to obtain the spatial distribution of the resistivity of a tissue [1]. Many applications have benefited from the ERT technique due to its low cost, high speed and non-invasive advantages. The implementation of ERT requires a conductive domain where electrodes are required to be directly attached to its boundary. An electric field is generated from injecting the current via electrode patterns where an Alternating Current (AC) source would be required. For voltage measurement, voltmeters would be applied to electrodes simultaneously. The measurement strategy could be selected from among the neighboring method, opposite method, adaptive method, etc. [2]. Typical ERT problems would normally begin with a forward problem, where distribution of the potential could be worked out using a simulation modeling tool. The sensitivity distribution of the whole domain could be calculated via the perturbation method [3]. Then, the conductivity distribution could be recovered via an inverse problem solver, which is known as an algorithm to reconstruct images from measured real boundary data.

The inverse problems of electrical tomography are actually ill-posed, and regularization methods, in this case, would be very important for recovery of conductivity mapping. Methods using the strategy of least squares solution, such as, Tikhonov regularization, are quite popular. However, most algorithms would give a result with blurred edges of the object boundary, and lead to reconstruction error because Tikhonov would over-smooth the images. In the past years, another method called total variation (TV) with different TV functions, such as, [4,5] has been proposed. TV has attracted attention, as higher-quality images can be obtained.

According to dynamic ERT cases, most of the traditional ERT algorithms reconstruct static images using individual frames of data which assume that no correlations exist between adjacent frames, such as, Tikhonov [6], Gauss-Newton one-step [7], and total variation [8] etc. However, to reconstruct images with the regularization methods that working frame by frame may not be an optimal choice, and would result in overlapping of artefact images, although only a few papers proposed methods that account for the temporal correlation effect. High temporal resolution is one of the advantages of the ERT system, and information on the correlation between individual frames should be explored to contribute to the image quality. A few types of algorithms account for the temporal correlation effects. First, Kalman filtering has been used in different tomographic techniques. For example, M. Vauhkonen first proposed the Kalman filter method (1998) to track fast changes in electrical impedance tomography (EIT) [9]. More recent work regarding the Kalman filter in dynamic imaging fields was also proposed, for example, M. Soleimani and M. Vauhkonen [10] used the Kalman filter for electrical capacitance tomography (ECT) and electromagnetic induction tomography (EMT), and demonstrated the Kalman filtering approach could improve the spatiotemporal resolution (2007). A. Lehikoinen et al. (2009) evaluated dynamical conductivity distribution in a porous medium using the Extended Kalman Filter [11]. A. K. Saibaba (2014) tracked CO_2_ movement with a fast Extended Kalman Filter [12]. In addition, another algorithm known as the temporal one-step solver (TOS), which is based on the GN one-step method was proposed by A. Adler and T. Dai (2007) in [13], and has been investigated by comparing with the Kalman filter and conventional GN one-step method which work as if there are no correlations between successive frames. More recently, Yerworth and Bayford (2013) first proposed interpolating EIT measurements because conductivity changes during frame acquisition [14]. Gagnon, Hervé, et al. (2015) proposed comparison works to assess advantages and drawbacks of previously presented approaches using different types of data frames with three reconstruction algorithms [15]. Chen, Bo, et al. (2018) proposed a novel spatiotemporal total variation (STTV) method for assessing the performance of 2D and 3D moving objects using both simulation and experimental results [16]. Temporally linked algorithms such as the one shown in the above examples provide an opportunity for faster data collection and less averaging in ERT data. A smooth regularization, however, will limit the time resolution. A temporal TV with TV regularization in time can overcome this problem, providing both high speed and sharp temporal responses.

In practice, ERT could potentially be combined with a tomography-based control system. To reach the requirement of controlling the application, high-quality images from ERT would be needed, and useful information should be extracted based on these reconstructed results for the proposes of, for example, implementation of emergency operations to avoid undesirable conditions. In this paper, we are particularly interested in the temporal and spatial performance of the spatiotemporal total variation (STTV) method. By comparing it with the TOS algorithm, we want to explore spatiotemporal information along the time domain and assess the gradients and time response of both approaches. The results of this paper are based on 2D phantom experimental tests with dynamic movement of inclusion. For each set of results, STTV and TOS are employed using the same measured data set to ensure the consistency.

## 2. Method

ERT reconstructs images based on its boundary measurement data. Regarding a common ERT model, the conductive domain is normally bounded by electrodes, where the electric field is generated from injected currents. The participation of inclusion would change the distribution of the electric field, which would affect the boundary measurement data, and hence, conductivity distribution images would be affected. In terms of one injection between a pair of electrodes, the electric field is produced, and the generated voltage between other electrodes pairs could be measured and recorded via the ERT system as measured data. For a 16-electrode ring, there will be 208 individual measurement data points for both the background and object, where 208 corresponding voltage difference Δu values will be used as boundary data to reconstruct a frame of the image. In this paper, we test the performance of dynamic cases. The difference between the static and dynamic case is that the object keeps moving its position, which makes the electric field change distribution at the same time, where the effects of magnetic fields are not considered in order to simplify this physical model. Because the process of current injection as well as the conductive field remain, both cases would have the same forward model. However, the time step of data collection cannot be neglected in practical dynamic cases as the dynamic electric field would affect the data measurement although this could be very fast.

### 2.1. Forward Problem

For a specific region of ERT, the forward problem is to estimate the potential distribution based on the given applied current on electrodes, shape of the region, and a known conductivity. The forward solver mainly uses the finite element method (FEM), which divides the domain into many elements, and then we can work out the potential distribution from the values on the nodes between elements using a simulation tool. In this paper, the condition is under low excitation frequency, which creates the assumption for the physical model that the effect of magnetic induction could be neglected for such a pure resistive model in a quasi-static electric field. For an ERT domain Ω with its boundary dΩ, the current density is contributed by conductive current $J_{C}$ and the conductive current density $J_{s}$ which should remain at 0 since there is no source of the internal domain, and the governing formulation could be derived from the following equations [17]

(1)  ∇×E=0 ∇·J=0    J=σE E=−∇φ

where σ is conductivity, and E, φ donates the electric field and electric potential. The three equations are the charge conservation law, Faraday’s law and continuum version of Ohm’s law, respectively. With low frequency assumption in Maxwell’s equations ignoring the wave propagation effects, the ERT forward problem can be described by:

(2)  ∇·σ∇φ=0 

The current density j is generated while the current is injected sequentially via electrodes on the boundary of the domain; in this case, the boundary condition must be involved, with $\vec{n}$ representing the normal vector which gives:

(3) $$\;j=\sigma \frac{\partial \varphi }{\partial \vec{n}}\;$$

Regarding the electrode model, a complete electrode model (CEM), which combines the features from other previous models [18] was chosen and has the following formulations:

(4) $$\;u+z_{l}.\sigma \frac{\partial u}{\partial \vec{n}}=v_{l}\;$$

On the boundary, the current and voltage Kirchhoff laws must be satisfied:

(5) $${∯}_{d\Omega }j\cdot dS=0$$

(6)  ∮E·dl=0

In Equation (4), the expression displays the relationship of boundary potential $v_{l}$, the potential u on the electrode and the voltage drop on the contact impedance $z_{l}$ on each electrode. Note that the current density remains 0 on the gap (between electrodes). In order to get a unique solution, Equations (5) and (6) are involved here, which represent the conservation of charge and the voltage. Both equations could also be simplified as the sum of current/potential of all electrodes, which also equals zero.

To simplify the expression, a forward operator could be defined:

(7)  F(σ)=u 

A simplified equation could also be given by:

(8) Δu =J·Δσ+noise

where *J* donates the Jacobian matrix, defined as $\frac{\delta u}{\delta \sigma }$.

### 2.2. Inverse Problem

The Inverse problem of ERT is actually to obtain the conductivity distribution and reconstructed images, which requires the inversion of the formulation (8). However, the equation is actually non-linear, and the inverse problem of ERT is ill-posed, which means the existence, uniqueness, and the stability of the solution are not meet at the same time [19]. In this case, regularization methods are required for the optimization problem.

In the static ERT, the electrical conductivity can be described by σ(x,y,z), whist a time-varying conductivity in dynamical ERT means that the conductivity can be described by σ(x,y,z,t). In the actual ERT experiments, we are dealing with a discrete number of spatial and time steps, which can be described by spatial and time resolutions. In dynamic ERT, with moving inclusions, the assumptions of forward modeling Equation (2) are still valid, and conductivity changes from σ(x,y,z) to σ(x,y,z,t). Further extension of static inversion to temporal inversion is described in the following section.

In this paper, we discuss the cases of dynamic 2D ERT reconstruction. In this case, the expected conductivity difference would be a 3D object, which contains both spatial and temporal components. Comparison between Spatiotemporal Total Variation Algorithm (STTV) and Temporal one-step solver (TOS) will be made. Some results are displayed and discussed in Section 3 and Section 4.

#### 2.2.1. Spatiotemporal Total Variation Algorithm

The Spatiotemporal Total Variation Algorithm is based on the Split Bergman method, and has been used to reconstruct the images from a flow system in [16]. For solving an inverse problem, a penalty term could be added for optimization. The penalty term of a total variation problem is normally given by $\;G_{TV}=\alpha ||\nabla \Delta \sigma {||}_{1}$. STTV combined spatial and temporal TV functional, and the constrained problem of STTV is given by (9), and formulations (10) and (11) represent its iterative scheme:

(9) $$\;arg\;\underset{\Delta \sigma }{\min}{||\nabla_{x,y}\Delta \sigma ||}_{1}+{||\nabla_{t}\Delta \sigma ||}_{1}\;s.t.\;{||\tilde{J}\;\Delta \sigma -\Delta u||}_{2}^{2}\leq \delta \;$$

(10) $$\;\Delta \sigma^{k+1}=arg\;\underset{\Delta \sigma }{\min}{||\nabla_{x,y}\Delta \sigma ||}_{1}+{||\nabla_{t}\Delta \sigma ||}_{1}+\overset{I}{\underset{i=1}{\sum }}\frac{\mu }{2}\;{||\tilde{J}\;\Delta \sigma -\Delta u^{k}||}_{2}^{2}\;$$

(11) $$\;\Delta u^{k+1}=\Delta u^{k}-\;\tilde{J}\Delta \sigma^{k+1}+\Delta u\;$$

Due to the difficulty of solving a non-differential TV functional, auxiliary variables d*x*, d*x*, d*t* are involved here for applying the ‘splitting’, which is a similar to Split Bregman for splitting the data fidelity term and the non-differentiable l1-norm penalty term. The spatiotemporal component is included in STTV to correlate the consecutive frames, rather than recover Δσ individually:

(12) $$\;\left(\Delta \sigma^{k+1},dx,dy,dt\right)\;\;=arg\underset{\Delta \sigma ,dx,dy,\mathrm{dt}}{\min}{||(dx,dy)||}_{1}+{||dt||}_{1}+\frac{\mu }{2}\;{||\tilde{J}\;\Delta \sigma -\Delta u^{k}||}_{2}^{2}\;s.t.\;d_{i}=\nabla_{i}\Delta \sigma ,\;i=x,y,t\;$$

#### 2.2.2. Temporal One-Step Solver

Another algorithm called the temporal one-step solver (TOS) was proposed by [13] in 2007, which was based on the Gauss-Newton one-step algorithm. Instead of reconstructing images frame by frame, TOS uses a data set that combines the data of nearby frames of frame *n*, where the data set and the conductivity change could be given by:

(13) $$\;\tilde{\Delta u_{n}}={\left[\Delta u_{n-d},\;\cdots ,\;\Delta u_{n}\;,\ldots ,\;\Delta u_{n+d}\right]}^{T}\;$$

(14) $$\;\tilde{\Delta \sigma_{n}}={\left[\Delta \sigma_{n-d},\;\cdots ,\;\Delta \sigma_{n}\;,\ldots ,\;\Delta \sigma_{n+d}\right]}^{T}\;$$

where in (13) and (14), there are d frames of data before and after the frame *n*; in this case, the length of the data set would be (2*d* + 1), and d is an integer and must be smaller than *n*.

The forward problem could be modified into:

(15) $$\;\tilde{\Delta u_{n}}=\tilde{j}\tilde{\Delta \sigma_{n}}+\mathrm{noise}\;$$

Using the GN One-step method, the inverse problem would be defined as:

(16) $$\;{||\tilde{\Delta u_{n}}-\tilde{j}\Delta \sigma_{n}||}^{2}+\lambda^{2}{||\tilde{\Delta \sigma_{n}}||}^{2}\;$$

By solving (16), as stated in [12], the equation is written as:

(17) $$\;\tilde{\Delta \sigma_{n}}=\left[gamma⊗\left(PJ^{T}\right)\right]{\left[gamma⊗\left(JPJ^{T}\right)+\lambda^{2}\left(I⊗V\right)\right]}^{-1}\cdot \tilde{\Delta u_{n}}\;$$

In (17), V is an identical matrix, and equals $R^{-1}$ , where $R=\alpha R_{1}+\beta R_{2}+\gamma R_{3}$. $R_{1}$ is contribute to a NOSER prior, a diagonal matrix with its diagonal elements equivalent to the diagonal elements of $J\cdot J^{T}$. $R_{2}$ donates an identical matrix with the size of $R_{1}$.

## 3. Experiments and Results

### 3.1. Experimental Set up

For the data collection in the experiments in this paper a complete ERT system was required, which was composed of an ERT Hardware system, PC (with software) and a sensor. 

**Figure 1:**
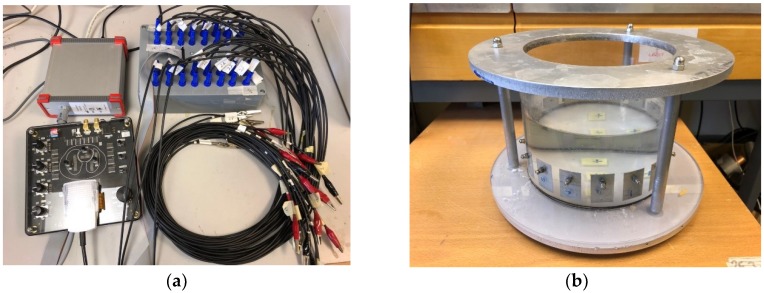
(**a**) Swisstom EIT Pioneer system; (**b**) experimental tank.

The hardware system used in this paper is known as the EIT Swisstom Pioneer system [20] with 32 channels (Picture of Swisstom Pioneer system is showing in Figure 1(a)), which has the following main components:

- 16 double-channel EIT chips to control 32 electrodes
- Smart SensorBeltConnector integrated with AC current injection (1–7 mA, 50 KHz–250 KHz), voltage signal demodulating, high speed data collection (up to 80 frames/s)
- Interface module: Frame synchronisation input and output, synchronisation signal, power management between SensorBelt and SensorBeltConnector
- Power supply
- Sensor

The 2D sensor used in the experiments is a cylinder-shaped PMMA container (as showing in the Figure 1b), with a diameter of 19 cm and height of 25 cm. On the side wall of the sensor, 16 electrodes (2 cm × 4 cm) are evenly fixed along the surface. A heavy circular metal board sits on the top of the container, connected by three long screws with the base board, in order to avoid the leaking of any liquid.

The STEM data collection software runs with the Swisstom EIT Pioneer to collect data, where the excitation frequency, current peak value, data collection speed and current pattern can be adjusted. In addition, the connection with electrode could easily be checked via the Sensor Quality panel, and a real-time dynamic image is also available.

### 3.2. Experimental Tests

In this section, we show reconstructed images of different tests. Tap water and a plastic bar (3.1 cm diameter) were used as the background and the moving object, respectively, under test conditions. Cross movement and circular movement were considered as two types of movement in the dynamic test. In all of the experimental tests, the peak value of the exciting current used was 7 mA, and the operation frequency was 270 KHz. To compare the two algorithms, a considerable data was collected at different frequencies on various dynamic cases for image reconstruction. In this section, we only displayed some typical results. Different dynamic movement types were set up, and results from each type of dynamic movement using both algorithms are displayed. Regarding the image reconstruction, 6 images were extracted from the generated dynamic image of each movement type to demonstrate the performance of both algorithms.

#### 3.2.1. Dynamic Test

**Figure 2:**
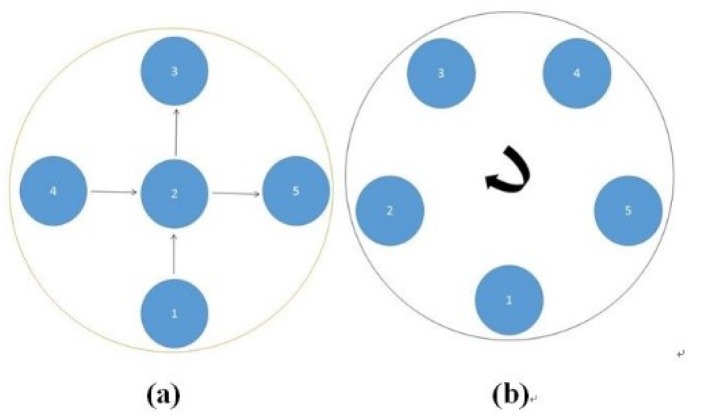
Illustration of the dynamic movement type. The cross movement is shown in (**a**); and (**b**) illustrates the circular movement.

The dynamic test is divided into two types of movement in this paper: cross movement and circular movement. Cross movement is the case where the inclusion moves cross the domain through the center along the diameter from one side to the other, and the circular movement where the object moves along a circle near the boundary of the domain. Illustrations of the dynamic movement can be seen in the Figure 2.

#### 3.2.2. Test 1 Cross Movement

In the first dynamic test, the plastic bar was driven manually to move from the bottom to the top, then from left to right across the domain. As shown in Figure 2 (a), the inclusion movement was across the domain from 1 to 3 via position 2 in the centre; then the inclusion moved from 4 to 5. The excitation current was 7 mA with a frequency of 270 kHz, and the data collection rate was 24 frames/s. In both tests of the cross movement, 850 and 950 frames of measurement data were collected with background data included. The measurement data from the background (with tap water only) was more than 100 frames when there was more than 5 s before starting to put the plastic bar into the tank, so the average background data of the first hundred frames would be used for the purpose of removing some noise. By using the STTV and TOS algorithms, reconstructed images were produced, as displayed in the table below, where the second column of the table shows the images of the TOS algorithm, and results from STTV are placed in the third column. Six slices of images have been included in the Table 1 and Table 2 to demonstrate the recovery images of the movement process using both algorithms.

From the results displayed in both tables, it was found that the moving process was monitored successfully and remained consistent with the movment of the plastic bar within the tank. Regarding the quality of reconstructed images, those from STTV have a sharp object boundary and are very smooth inside the object and in the background area, while TOS has blurred object edges. In terms of the performance of the temporal domain, many previous studies used these methods to produce dynamic images with individual frames of data. However, what can be seen from the table is the object is consistent in terms of its shape with both algorithms without any stretching along the movement direction.

#### 3.2.3. Test 2 Circular Movement

The implementation of circular movement is similar to Test 1, with the same excitation current value and frequency being employed. A data collection speed of 50 frames/s was used for the boundary data measurement. The bar moved clockwise along the circle close to the boundary and started in the location at the bottom shown in Figure 2b. In total, 530 frames of the boundary were collected with about 150 frames of background data. In terms of the implementation of using the STTV algorithm, it is worth pointing out that it was important to make sure that the background data was not included. For example, if the first 10 or 20 frames were still the background data, more noise would be introduced, which would integrate useless information and degrade the image quality, as STTV uses the time gradient to correlates each frame. Reconstructed images from STTV and TOS are compared in the Table 3.

The reconstructed images in this type of movement are quite stable compared with the result from the cross movement test. From these pictures, STTV still shows a better sharpness and a more smooth background than TOS.

In conclusion, both algorithms showed good performance in the dynamic test regarding the images. In comparison, images reconstructed using STTV are of better quality due to being sharper and having less noise. However, the discussion in this chapter is based on images only. To further support the good performance of STTV, some parameters pertaining to exploiting spatial and temporal information will be calculated, and some quantitative analysis based on these calculations will be carried out. 

## 4. Analysis and Discussion

As shown in the last chapter, images generated from the TOS algorithm suffer slightly from a blurred boundary of the recovered object, but STTV can produce higher-quality images, as those images benefit from higher sharpness and less noise. To analyse the advantages and drawbacks of both algorithms, in this section, some quantitative information is extracted and displayed to compare the two algorithms. The analysis is based on calculated results of the gradient, where spatial and temporal gradients are discussed separately. The response times are defined and worked out for both methods in order to further demonstrate how both algorithms contribute to the performance of the time domain.

### 4.1. Definition of Gradients and Time Response

#### 4.1.1. Spatial Gradient

The spatial gradient of an image refers to how the conductivity changes in space, which would normally be calculated along a direction (*x*, or *y*). The *x*/*y*-gradient could also be understood as the change of the slope value of the spatial distribution along the *x*/*y* direction. For image *u*, the gradient of *x* and *y* direction could be calculated by:

(18) $$\nabla_{x}u=\frac{\Delta u}{\Delta x}\;\left(S/m\right)\;$$

(19) $$\nabla_{y}u=\frac{\Delta u}{\Delta y}\;\left(S/m\right)\;$$

where $\nabla_{x}u$ and $\nabla_{y}u$ are spatial gradients of the respective x and y direction, and Δu is the conductivity step-change between 2 pixels. u is a slice of an image with 51 by 51 pixels, and p=51. In this paper, the magnitude of the gradient was calculated, which is given by:

(20) $$\nabla_{\mathrm{xy}}u=\sqrt{{\left(\nabla_{x}u\right)}^{2}+{\left(\nabla_{y}u\right)}^{2}}\;\;$$

#### 4.1.2. Temporal Gradient

Temporal imaging is defined as dynamic image reconstructions along the time series. A time gradient is useful for determining the dynamic performance as it describes the step changes of the whole variation process. The time gradient of the dynamic image could also be explained as the variations of conductivity in a pixel in the time domain. The dynamic process of the object could react to the temporal change in pixel values.

If we extract pixel values along the time sequence, the conductivity change in this pixel would be plotted as a 1-D line graph, where the pixel value variation illustrates the circumstance of the object movement. The temporal gradient could be defined as how much variation has been made in each time step with the time sequence, which is also a discrete gradient as the data set is combined with individual frames of data, although they are time correlated. The time gradient is calculated as:

(21) $$\;\nabla_{t}u=\frac{\Delta u}{\Delta t}\;\left(\frac{S}{m}/s\right)$$

#### 4.1.3. Time Response

The time response is defined by how fast the algorithm is capable of reacting to temporal change, which could be used to determine the dynamic performance of the result quantitatively. Regarding the temporal change in a pixel where the inclusion occurred during the moving process, the algorithm that shows faster response would be an optimal choice. The expression of the Time Response could be given by a time interval that corresponds to where the conductivity decays from the background value to the peak value.

### 4.2. Spatial and Temporal Gradients

#### 4.2.1. Spatial Imaging

In Table 4, Table 5 and Table 6, there are few items displayed: images of the spatial gradient, 1-D plots of spatial distribution and the corresponding plots of the spatial gradient. There are 3 tables in this part, where images of corresponding 3 positions are extracted for analysis.

For an ideal case, the boundary of the object is supposed to be clear, so the spatial distribution of the object boundary is expected to be sharp, and the whole object area and also the background should be retained at the corresponding conductivity values. A 1-D Plot of the spatial distribution of a perfect ideal case would show a square-shape wave, with the result that only two sharp changes would be seen between the object and background. Some comparisons were made between the STTV and TOS algorithms, displayed in Table 4, Table 5 and Table 6. 

From the images of the spatial gradient across three different positions (the data used in this part are consistent with the tests in the last section) within the tank; regarding the results using TOS, the object area is unevenly distributed in the images of the spatial gradient distribution. However the results from STTV show a very clear object boundary. The comparison of the conductivity distribution is given by the 1-D plot in the third row of each table, where images have been normalized in order to make the contrast. The blue and red colored lines in the plotted line graphs indicate the result recovered from STTV and TOS, respectively, regarding the spatial distribution and gradient changes. It can be seen from the conductivity distribution using TOS that the lowest values are always in the middle of the wave, and the value of conductivity drops down gradually and comes back up slowly again, which indicates an unclear boundary between the background and the object, as it would be hard to recognize the exact correspondence to the boundary. The plots from STTV are given by nearly square-shape waves. The plotted line remains flat either in the background or the object area, and a very shape change is observed between the background and the target. The last graph shows the comparison of the spatial gradient, in which the plots of STTV always display two sharp changes that have the same wave shape of the plot from the true image, but the gradient plot of the TOS result is deformed due to the lower sharpness of the recovered target. 

#### 4.2.2. Temporal Imaging

Reconstructed results from cross and circular movement tests (images shown in the last chapter) using TOS and STTV are displayed in this part for analyzing the spatiotemporal performance. First, spatial and temporal gradients are displayed and compared. In addition, the temporal variation of both dynamic settings using two different algorithms was taken into account to discuss the time response. All results regarding two different types of movement are showing in Table 7 and Table 8.

In fact, the spatial gradient is the change between the current pixel value and the next neighboring pixel value along the defined direction, whilst the temporal gradient is the variation between two adjacent frames (variation along the time series). The images of gradients in this section are based on a random frame *N* during the dynamic process, so the spatial gradient would be from the reconstructed image using frame *N*, and the temporal gradient would be the difference between image number *N* and (*N* + 1). According to the results of gradients, in terms of the spatial domain, obvious changes in the conductivity distribution along *x*, *y* directions could be observed with a clear boundary between the background and inclusion. On the background or inside the object area, the conductivity variation between consecutive pixels is very small as it could be seen that the spatial gradient in these area tends to 0. By comparison with TOS, results show that the object is observable and recognizable; however, the conductivity value keeps changing inside the object in both directions, and the lower sharpness of the boundary indicates less sharp variation between the background and the inclusion. 

Regarding the temporal change between adjacent frames, relatively small variation between consecutive frames should be shown in the temporal domain due to the high data collection speed employed. In this case, very little shift is expected on the object boundary, and almost zero change should be detected in the rest of the domain as the consecutive frame data should be very similar. As shown in the figures of both dynamic movement types, the STTV results are proved to have higher immunity to the noise, and the changes in the time domain of STTV result is more consistent compared with the results of TOS. In terms of the decay of the absolute spatial/temporal gradient based on the gradient images in the second row of the table, the line graph of the result using STTV shows faster decay than the one using the TOS method. The plot of decay displays the extent of the variation from the maximum value to 0, which numerically indicates the performance of the spatial and temporal change between neighboring frames. This result quantitatively demonstrates that STTV would potentially reconstruct high-quality images of the dynamic case.

### 4.3. Time Response

To further illustrate the performance in the time domain, the conductivity variation of pixels along the time series was emphasized and further studied with respect to the time response. During the dynamic process, when the inclusion arrives at and then leaves a pixel, a dynamic change in such pixel would be generated. For a tomography-based control system, it is quite important whether an algorithm could react to a moving inclusion, as a timely response is definitely required for a making decision and implementing action timeously.

**Figure 3:**
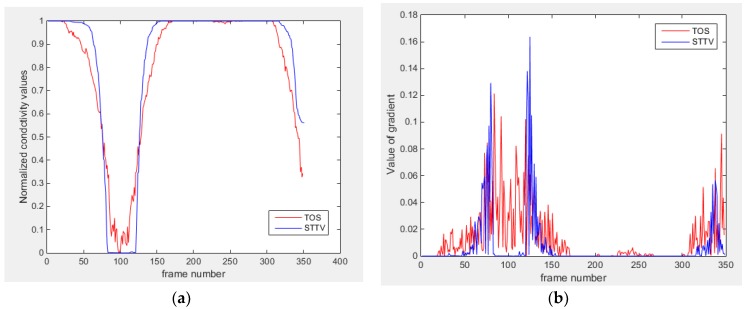
(**a**) Time variation of a pixel extracted from the results of the cross movement; (**b**) the plot of the corresponding temporal gradient. The red line is the result when using TOS, and the blue line indicates the STTV results.

**Figure 4:**
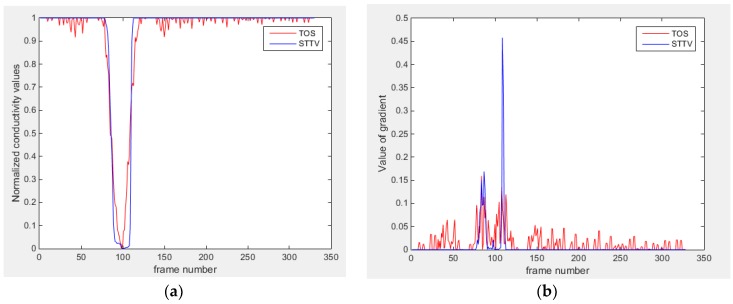
(**a**) Time variation of a pixel extracted from the results of the circular movement; (**b**) the plot of the corresponding temporal gradient. The red line is the result using TOS, and the blue line indicates the STTV results.

Based on the results from two dynamic movements using both algorithms, conductivity changes along the time series from selected pixels as well as the corresponding temporal gradient are shown in Figure 3 and Figure 4. From the time variation plots of both results, it is obvious that they display how fast the conductivity varies from a maximum value (corresponding to the background conductivity) to nearly zero and back up again, where 0 indicates when the object went through the pixel. In comparison, STTV would take slightly less time. Numerical calculations of the response time of both algorithms were worked out, and displaying in Table 9 and Table 10. In the cross movement, the displayed results were from the data collection speed of 24 frames/s. The response times of both algorithms, in the case where the object enters the pixel, were 2.12 s and 3.21 s, and in the other movement, the frame speed of 50 frames/s was used, and STTV showed a faster response than TOS. The results stated above indicate STTV had a faster response to the conductivity change in the time domain.

## 5. Conclusion

Dynamic imaging is a very important topic to be investigated due to the fact that there are many requirements regarding moving objects inside the bounded domains in real life for monitoring and systematic controlling proposes. ERT benefits from its high temporal resolution, and would potentially have temporal information exploited along the time domain. In terms of the reconstruction algorithms, the ones using individual frame data are not optimal, as they ignore the dynamic change and the variation between each time steps could not be considered properly. 

In this paper, we investigate the performance of 2D dynamic movement using experimental data. To evaluate both the spatial and temporal performance of the STTV algorithm, results from using both the STTV and TOS regularization methods are displayed for comparison. In the spatial domain, both algorithms show good performance. Through the analysis of the dynamic experimental results, it could be concluded that STTV generates sharper and less noisy images, However, optimization of the parameter selection is still required to be studied in future work, and it would have potential to be upgraded. A sharp image would be preferred, as the boundary of recovered images could be detected easier than blurred ones. Performance in the time domain of both methods was compared using the temporal gradient and response time, where STTV showed a faster response time with respect to the dynamic change of conductivity based on the quantitative calculation of the time response of different dynamic movements. This discovery is useful when it will be used in real applications of tomography-based control systems in the future.

## Figures and Tables

**Table 1:** Reconstructed images of cross movement in Test 1, where the inclusion moves from the bottom to the top.

No.	TOS	STTV
1	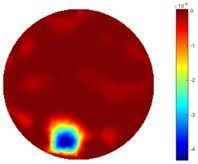	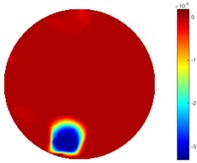
2	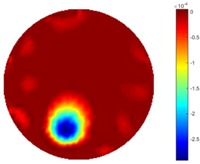	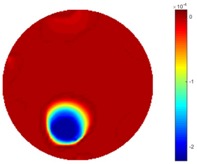
3	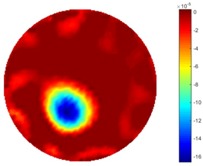	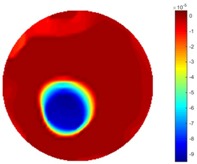
4	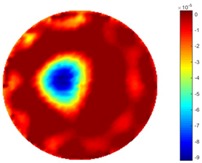	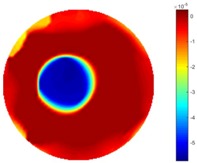
5	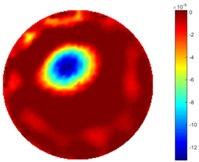	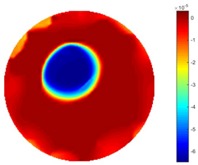
6	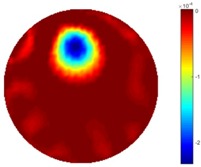	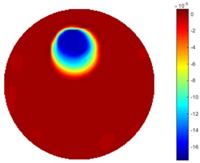

**Table 2:** Reconstructed images of cross movement in Test 1, where the inclusion moves from the left to the right hand side.

No.	TOS	STTV
1	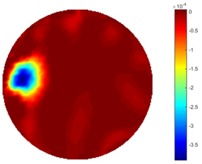	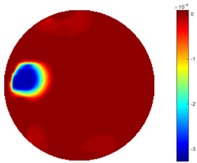
2	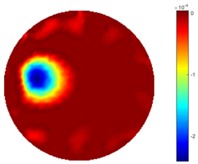	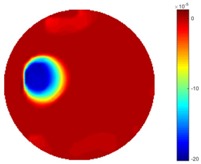
3	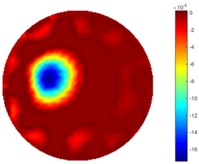	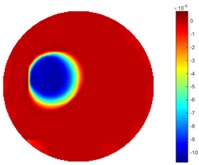
4	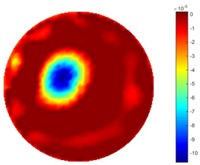	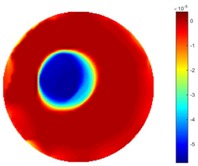
5	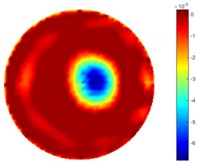	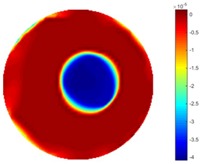
6	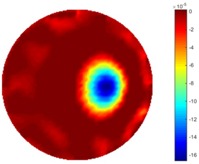	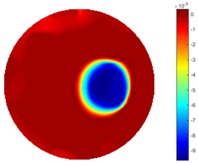

**Table 3:** Reconstructed images from the circular movement test.

No.	TOS	STTV
1	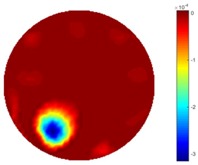	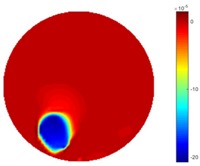
2	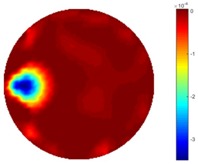	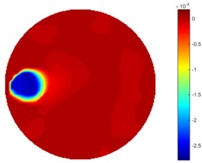
3	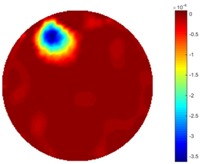	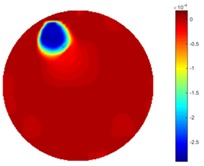
4	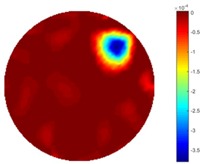	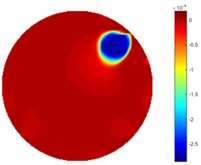
5	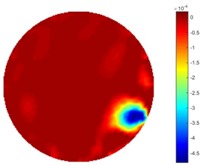	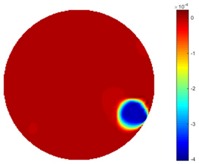
6	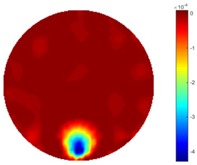	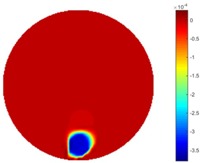

**Table 4:** Spatial gradients of the results produced from STTV and TOS algorithms, where the reconstructed object is near the center. The image of the spatial gradient is generated from calculating the gradient of the reconstructed image. The spatial distribution plotted in the second row uses the middle row of the image matrix, and the 1-d plot of the spatial gradient is produced to evaluate the spatial variation.

Algorithm	TOS	STTV
Spatial gradient (S/m)	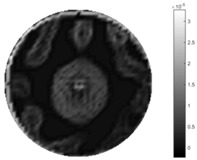	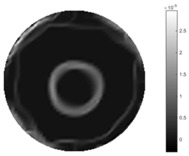
1-d plot of spatial distribution	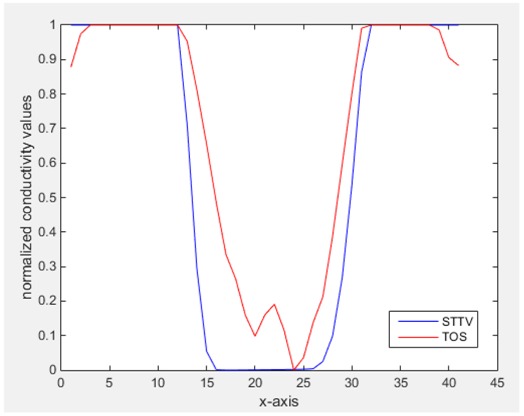	
1-d plot of spatial gradient	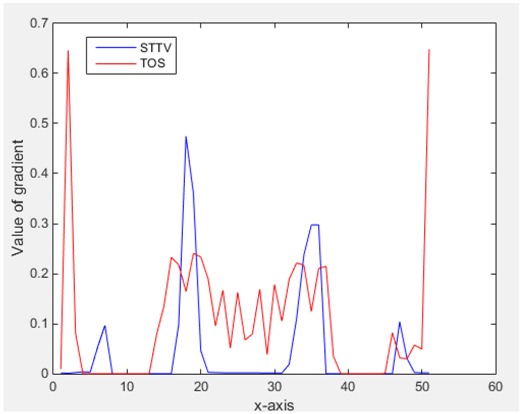	

**Table 5:** Spatial gradients of the results produced from STTV and TOS algorithms, where the reconstructed object moves to the second position.

Algorithm	TOS	STTV
Spatial gradient (S/m)	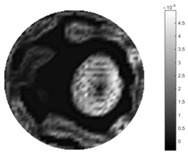	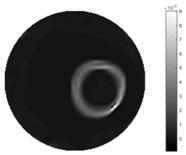
1-d plot of spatial distribution	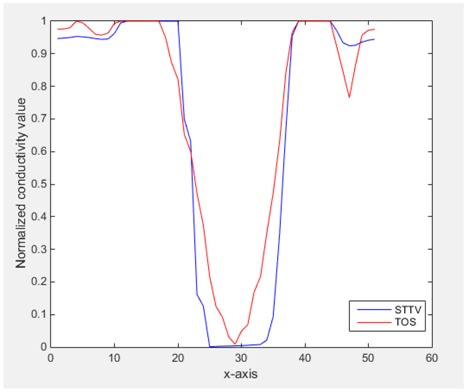	
1-d plot of spatial gradient	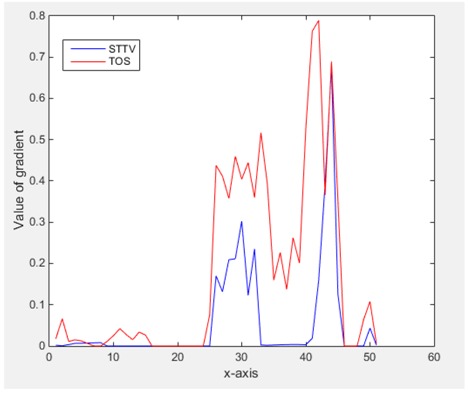	

**Table 6:** Spatial gradients of the results produced from STTV and TOS algorithms, where the reconstructed object moves to the edge of the domain.

Algorithm	TOS	STTV
Spatial gradient (S/m)	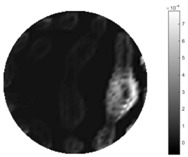	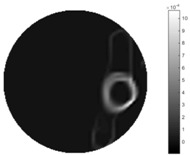
1-d plot of spatial distribution	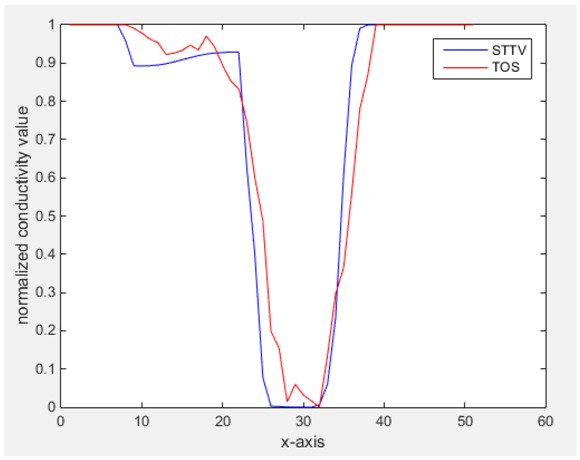	
1-d plot of spatial gradient	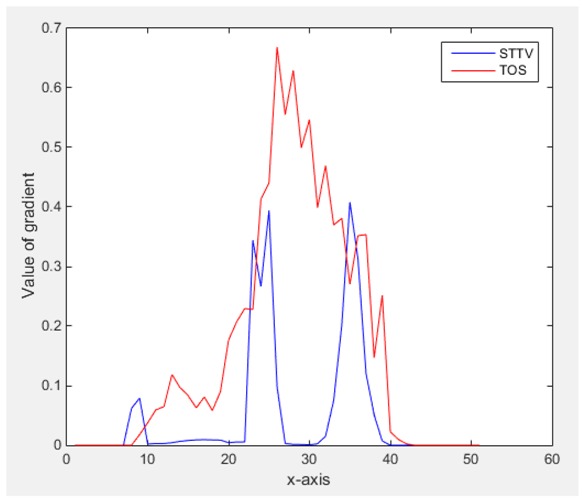	

**Table 7:** The results of spatial and temporal gradients of the circular movement test using STTV and TOS algorithms. The image of the spatial gradient is generated by calculating the spatial gradient of the reconstructed image of a specific frame number, and the temporal gradient image is based on the time gradient between neighboring frames. The line graph of the decay coefficient is based on the gradient value from the images of spatial and temporal gradients.

	Spatial Gradient (S/m)	$\mathrm{Temporal}\;\mathrm{Gradient}\;(\frac{S}{m}/s)$
TOS	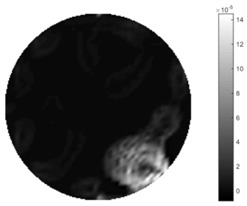	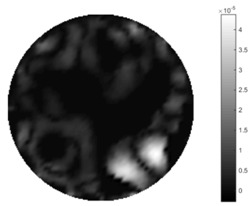
STTV	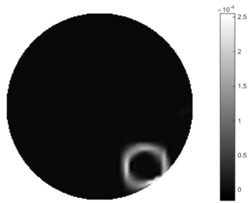	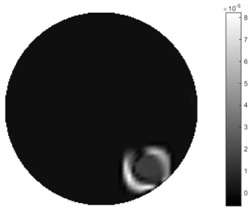
Decay of coefficients in spatial	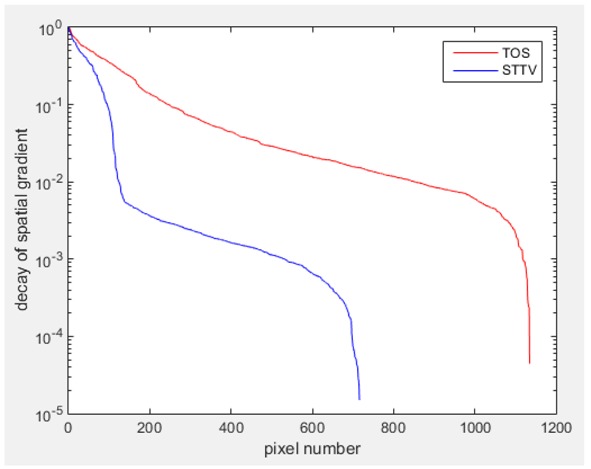	
Decay of coefficients in time	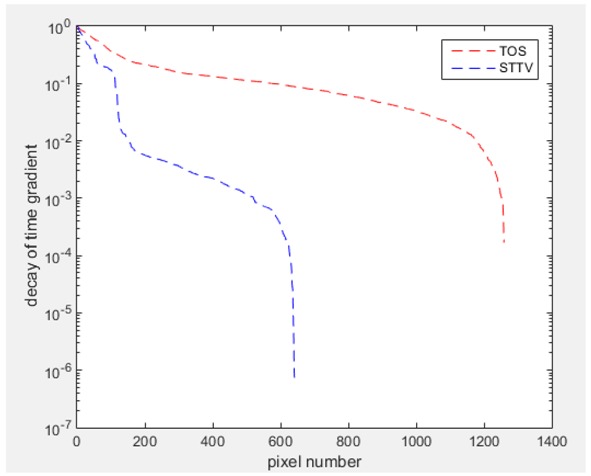	

**Table 8:** The results of spatial and temporal gradients of the cross movement test using STTV and TOS algorithms. The image of the spatial gradient is generated by calculating the spatial gradient of reconstructed images of a specific frame number, and the temporal gradient image is based on the time gradient between neighboring frames. The line graph of the decay coefficient is based on the gradient value from the images of spatial and temporal gradients.

	Spatial Gradient (S/m)	$\mathrm{Temporal}\;\mathrm{Gradient}\;\;(\frac{S}{m}/s)$
TOS	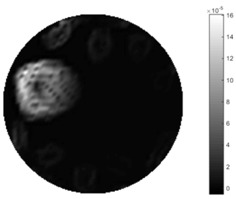	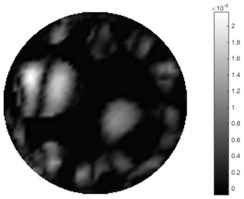
STTV	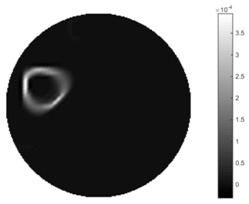	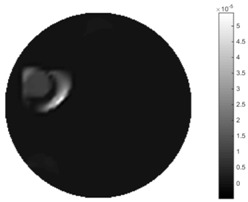
Decay of coefficients in spatial	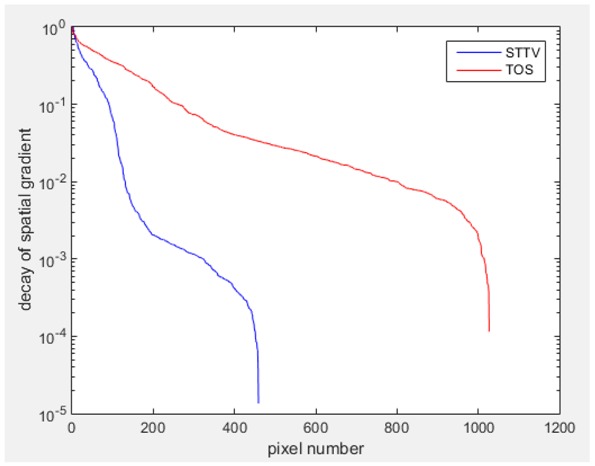	
Decay of coefficients in time	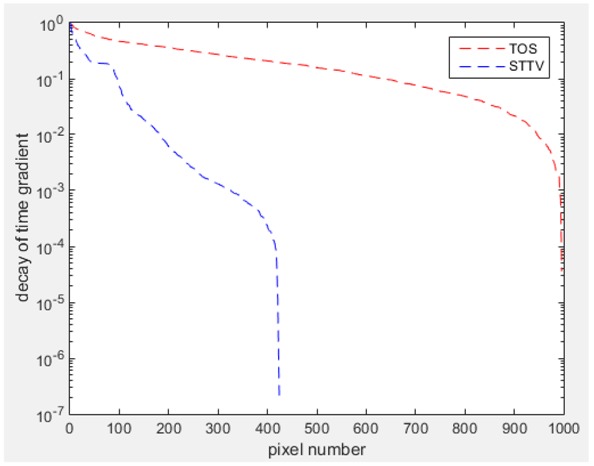	

**Table 9:** Time response of both algorithms when testing the cross movement.

Algorithm	TOS	STTV
Time response	3.21 s (77 frames)	2.12 s (49 frames)

**Table 10:** Time response of both algorithms when testing the circular movement.

Algorithm	TOS	STTV
Time response	0.52 s (26 frames)	0.26s (13 frames)
